# A Multimodal, Electrocardiography-Centric Clinical Data Platform With Artificial Intelligence-Assisted Querying for Cardiovascular Research

**DOI:** 10.7759/cureus.115479

**Published:** 2026-08-31

**Authors:** Carlos Rodrigo Rivero, Vicente Gallego Sánchez, Alejandro Cortés Beringola, José Ángel Cabrera Rodríguez, Pedro Martínez Losas

**Affiliations:** 1 Information Technology, Spika Tech, S.L., Madrid, ESP; 2 Research, Spika Tech, S.L., Madrid, ESP; 3 Cardiology, Hospital Universitario Infanta Leonor, Madrid, ESP; 4 Cardiology, Hospital Universitario Quirónsalud, Madrid, ESP

**Keywords:** cardiovascular informatics, clinical data integration, electrocardiography, large language models, natural language interface, text-to-sql

## Abstract

Objective: Large-scale electrocardiographic (ECG) datasets are increasingly available but remain fragmented, poorly integrated with patient-level phenotypic data, and difficult for clinicians to query without programming expertise. We present VRCardio-Helper-Database, a unified clinical data platform that integrates ECG recordings from nine sources with patient demographic and anthropometric information and exposes an artificial-intelligence-assisted natural-language querying interface, built on a Model Context Protocol server, that translates clinical questions into safe, read-only database queries.

Methods: The integrated repository comprises approximately 1,185,800 ECG records from approximately 432,000 patients, with derived signal-processing metrics for the standard 12-lead sources and, for the 46-record in-house VRCardio-Explore cohort only, simultaneously acquired anthropometric measurements and electrocardiographic imaging signals. We evaluated the platform across five dimensions: dataset characterization; semantic accuracy of natural-language querying against a 40-query benchmark with consensus ground-truth Structured Query Language (SQL); robustness to linguistic variation; representative clinical use cases; and an expert clinical assessment.

Results: Query interpretation achieved 90% precision (95% confidence interval (CI), 77-96) and 98% recall (95% CI, 87-100), with a mean intersection-over-union of 0.97 across five reformulation clusters. Two senior cardiologists posed 35 free-form research queries, of which 28 returned a response (an 80% response-completion rate; the remaining 20% timed out), and rated the returned responses highly for medical correctness and interpretability (pooled overall quality 7.6/10; range, 6.9-8.4); because these ratings are computed only over responded queries, they should be read as an upper bound on real-world usability. Error analysis found that the few failures were associated with identifiable schema and terminology mismatches (unit conventions, dataset-specific diagnostic code strings, and cross-source synonyms).

Conclusions: By reducing the programming expertise needed to work with multimodal ECG data and enabling intuitive access, the platform may facilitate hypothesis generation in cardiovascular research. Given the modest evaluation size (a 40-query benchmark and 28 rated expert responses), a subset of queries that returned service-availability timeouts, and data-quality caveats in some derived metrics, these results should be read as an initial, carefully curated evaluation, and larger prospective multicenter validation is still needed.

## Introduction

Electrocardiography (ECG) remains one of the most widely used and cost-effective diagnostic tools in cardiovascular medicine. Its noninvasive nature and broad applicability make it a first-line tool for detecting and monitoring cardiac conditions such as arrhythmias, conduction abnormalities, and ischemic episodes. In recent years, the growing availability of large-scale digital ECG datasets has enabled significant advances in computational cardiology, particularly in the development of machine learning models for automated diagnosis and risk stratification.

Several publicly available datasets, such as Physikalisch-Technische Bundesanstalt XL (PTB-XL) [1], have contributed substantially to the field by providing extensive collections of annotated ECG records, facilitating reproducible research and the comparative evaluation of algorithms across a variety of clinical tasks. More recent initiatives have expanded these datasets by incorporating ECG-derived features and diagnostic claims, further enhancing their utility for machine learning applications [2]. Large-scale clinical repositories such as Medical Information Mart for Intensive Care IV - Electrocardiogram (MIMIC-IV-ECG) [3] have likewise provided access to hundreds of thousands of ECG records, supporting population-level analyses and the development of reliable models.

Despite these advances, existing ECG datasets have several limitations. First, they are often isolated repositories, each with its own data format, annotation scheme, and population characteristics, which makes cross-dataset analysis difficult. Second, they often lack detailed phenotypic information at the patient level, particularly anthropometric measurements and detailed clinical context, which are essential for understanding the relationship between electrophysiological signals and individual patient characteristics; even recent multimodal extensions focus primarily on combining signals with images or textual interpretations rather than integrating physiological data with the patient phenotype. Third, accessing these datasets often requires technical expertise in data engineering and Structured Query Language (SQL), which acts as a barrier for many clinicians and limits their direct involvement in data-driven research.

Efforts to standardize and ensure the interoperability of clinical data have led to common data models based on electronic health records, such as the Observational Health Data Sciences and Informatics common data model [4]. While these approaches improve data harmonization across institutions, they are not designed for high-resolution physiological signals such as the ECG, nor do they typically incorporate signal-processing features as first-order entities, nor do they address the need for intuitive interfaces that allow clinicians to explore complex datasets without advanced technical skills.

Recent advances in large language models (LLMs) [5] have opened new possibilities for interacting with structured data via natural language [6]. These systems can translate user intent into executable queries, enabling a shift from syntax-based to semantics-based data access. However, their application in clinical settings remains limited [7], particularly in fields requiring precise semantic interpretation and strict security constraints, such as cardiology.

Concurrent efforts to enable natural-language access to ECG data have focused primarily on signal-level question answering: Electrocardiogram Question Answering (ECG-QA) [8] introduced the first question-answering benchmark designed for ECG analysis, coupling PTB-XL records with question templates covering Standard Communications Protocol (SCP) codes, conduction intervals, and arrhythmia types; Q-HEART [9] subsequently proposed a multimodal LLM pipeline combining a deep ECG encoder with retrieval-augmented prompting over clinical reports. While these systems demonstrate the feasibility of LLM-driven ECG interpretation at the signal level, they address a fundamentally different task from clinical database querying: they answer questions about what a single ECG shows, not which patients in a large population meet a set of clinical research criteria. Accordingly, they operate on single-dataset benchmarks, require training of specialized multimodal encoders, and do not support cross-source cohort selection, population-level statistical analysis, or integration with patient-level phenotypic data. In the broader biomedical text-to-SQL domain, BiomedSQL [10] shows that even frontier models achieve only 58%-62% execution accuracy on scientific biomedical knowledge bases, confirming the difficulty of domain-specific natural-language querying and motivating domain-adapted system design.

There is therefore a clear unmet need for integrated, multimodal clinical data platforms that combine ECG signals, derived electrophysiological parameters, and patient characteristics within a unified architecture, while offering intuitive, natural-language access mechanisms tailored to clinical users.

We present VRCardio-Helper-Database, a unified ECG-centered clinical data platform that integrates heterogeneous data sources with patient demographic and anthropometric information and introduces an artificial intelligence (AI)-assisted natural-language querying interface, allowing users to express clinically meaningful queries without expertise in SQL. An end-to-end pipeline handles ECG ingestion, standardization, and automated feature extraction across heterogeneous formats, and the system supports both retrospective research and prospective clinical workflows under pseudonymization for data privacy and regulatory compliance. Because clinicians can query the data directly and because electrophysiological and phenotypic variables are exposed together, researchers can formulate and test hypotheses without intermediary data engineering, and the platform can surface relationships not observable from ECG signals in isolation. This is especially relevant in personalized cardiology, where interindividual variability shapes disease manifestation and treatment response. This work is novel in three respects relative to prior work: it integrates nine heterogeneous ECG sources under a unified schema linking signals, derived metrics, and patient phenotype (unlike single-source ECG repositories); it answers population-level cohort-selection queries across sources without ECG-specific training (unlike signal-level systems such as ECG-QA and Q-HEART); and it is a production-deployed clinical querying system with domain-adapted Model Context Protocol (MCP) tool descriptions (unlike general biomedical text-to-SQL benchmarks). Accordingly, our primary objective is to test whether an MCP-based natural-language interface can translate clinical research questions into correct database queries over this integrated repository. We assess this along five prespecified dimensions: 1) dataset characterization, 2) semantic accuracy against a benchmark with consensus ground-truth SQL, 3) robustness to linguistic variation, 4) representative clinical use cases, and 5) an expert clinical assessment.

## Materials and methods

System overview

VRCardio-Helper-Database is a pseudonymized clinical ECG repository that consolidates recordings from multiple independent research datasets, including PTB-XL [1], the Shaoxing-Chapman dataset [11], and MIMIC-IV-ECG [3], as well as an in-house cohort (VRCardio-Explore). The architecture follows a modular design integrating structured clinical data, raw signal storage, and an application layer that enables both programmatic and AI-assisted access (Figure 1). The system consists of three main components: a relational database for structured data, an object storage system for ECG signals, and an application layer that exposes RESTful services and natural-language querying. It is designed to support both large-scale retrospective research (bulk querying and metric computation) and prospective clinical use (ECG uploads by medical users).

**Figure 1:**
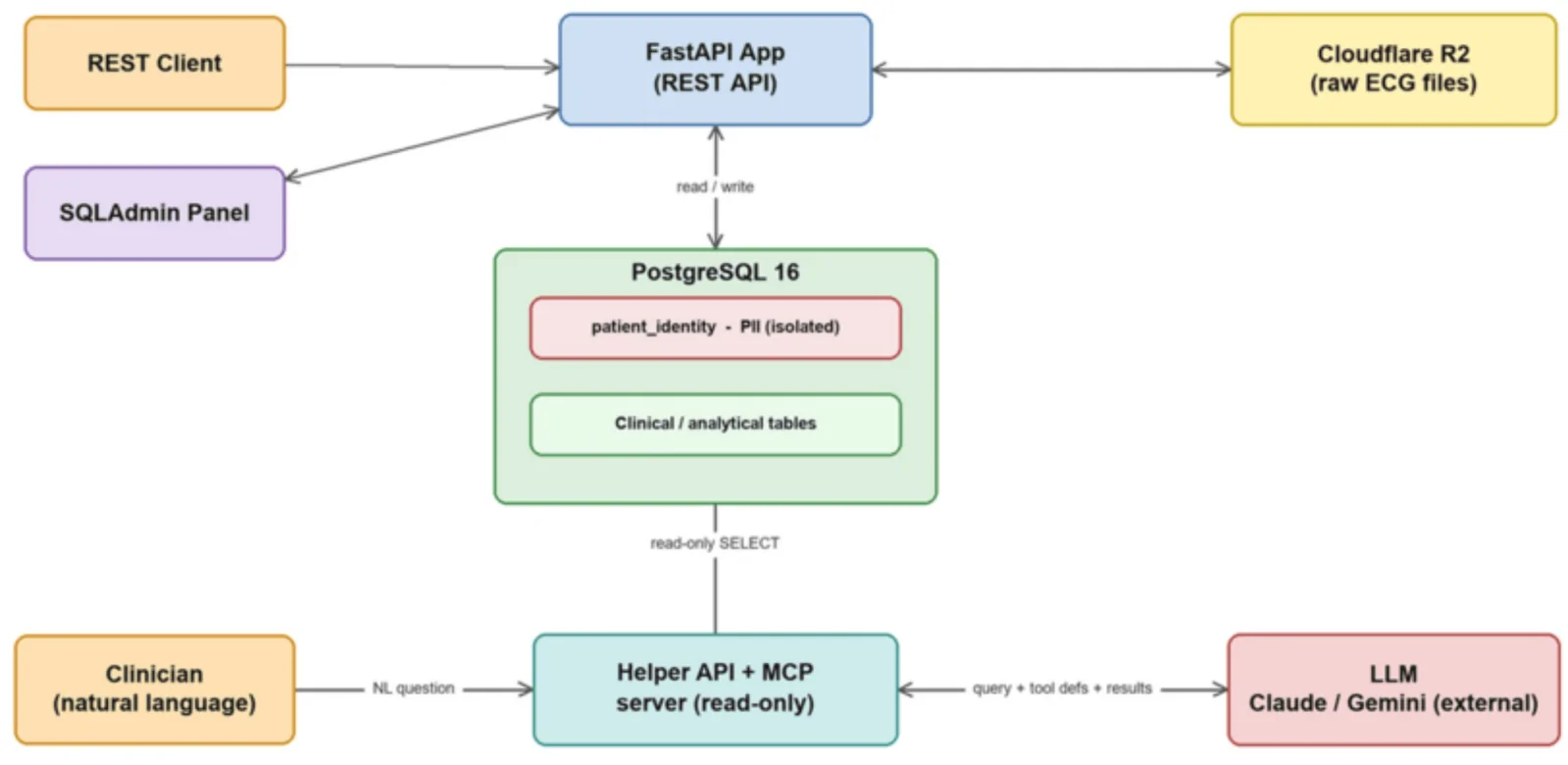
High-level system architecture of the VRCardio-Helper-Database platform System architecture and data flow of the VRCardio-Helper-Database platform. A REST client and an administration panel (SQLAdmin) communicate with the FastAPI application, which reads and writes a PostgreSQL 16 relational database and Cloudflare R2 object storage (raw ECG signal files). PII is isolated in a separate patient_identity table, apart from the clinical/analytical tables. In the natural-language querying path, a clinician's question is processed by the Helper API and MCP server, which sends the question, the MCP tool descriptions, and the read-only query results to an external LLM (Claude or Gemini); all model-generated SQL executes as read-only SELECT statements, and the MCP's only SQL tool rejects any write operation API, application programming interface; ECG, electrocardiography; MCP, Model Context Protocol; PII, personally identifiable information; REST, representational state transfer; SQL, structured query language; LLM, large language model Image credit: This figure was created by the authors using the Python Pillow (Python Imaging Library) imaging library (Jeffrey “Alex” Clark and contributors; open-source software)

Data storage layer

The system is built on FastAPI (Python 3.11; Python Software Foundation, Fredericksburg, VA) backed by PostgreSQL 16 (PostgreSQL Global Development Group, Berkeley, CA). Binary ECG files are stored in Cloudflare R2 (an S3-compatible object store; Cloudflare, Inc., San Francisco, CA), and the application is deployed on Fly.io (Fly.io, Inc., United States). The data model separates clinical data from personally identifiable information (PII), enabling pseudonymization and controlled access. The schema is organized into seven tables across two functional domains: a clinical core (patients, patient_identity, ecg_records, ecg_metrics, patient_measurements) and a user-management group (medical_users, user_patients). A complete entity-relationship diagram and full column-level schema are provided in the Appendix.

The patients table is the central entity, holding deidentified demographics, while PII is kept in strict isolation in patient_identity, linked by a unique 1:1 foreign key and populated only for VRCardio-Explore subjects to enable selective access control and future encryption-at-rest. The ecg_records fact table captures acquisition metadata, the Cloudflare R2 object key (null for the non-redistributed MIMIC-IV-ECG files), and an array of Standard Communications Protocol for Computerized Electrocardiography (SCP-ECG) diagnostic codes. The ecg_metrics table shares its primary key with ecg_records (1:0..1): for the seven standard 12-lead sources the parameters (heart rate; PR, QRS, and QT intervals; Bazett-corrected QTc; heart-rate-variability indices; signal-to-noise (SNR) ratio; and a binary arrhythmia flag) are computed from the raw waveform; for MIMIC-IV-ECG they are ingested from the source machine measurements (so SNR and heart-rate variability are unavailable); and for the 34-electrode VRCardio-Explore ECG-imaging (ECGi) recordings the 12-lead intervals are not computed, although every record still carries a metrics row. The patient_measurements table holds per-session anthropometrics (height, weight, diameters, somatotype, position, prior pathologies, and treatments) and is exclusive to VRCardio-Explore, while medical_users and the user_patients junction table implement role-based access control between clinicians and patients.

Application layer

The backend follows a decoupled three-layer architecture to ensure maintainability: a route layer that manages HTTP requests, authentication, and input validation; a data-access layer that implements database operations via an object-relational mapping (ORM) framework; and a model layer that defines the core data structures and business logic. A versioned REST API provides programmatic access to all system resources, including dedicated endpoints for data retrieval, complex filtering, and automated ECG ingestion; the complete catalog of 46 endpoints, grouped by resource, is provided in the Appendix.

AI-assisted querying layer

The system incorporates an AI-assisted querying interface built on an MCP [12,13] server, which translates user intent into structured, safe, read-only database operations for any compatible AI model. Rather than interpreting raw waveforms as in signal-level ECG question-answering systems [8,9], it operates exclusively on structured clinical metadata and precomputed signal features already stored in the database, enabling cohort selection and statistical queries across hundreds of thousands of records without retraining the ECG-specific model. The MCP layer exposes curated high-level tools rather than raw schema access [6,10], adding domain abstraction that reduces the surface area for query errors.

Security and privacy

To maintain clinical standards, the system enforces four safeguards. First, a strict separation of PII from clinical research data: PII is stored exclusively in the patient_identity table, isolated from all analytical tables. Second, role-based access control (RBAC) managed by the VRCardio platform. Third, read-only constraints for all AI-generated queries: the MCP server exposes no write, update, or delete tools, and its single SQL tool accepts SELECT statements only, rejecting INSERT, UPDATE, DELETE, and DDL at the application layer, so the model cannot modify the underlying data. The write-capable endpoints of the REST API (POST, PATCH, DELETE), used for administrative and clinical data management, belong to a separate application layer: the AI model can invoke only the registered read-only MCP tools and has no mechanism to issue REST API requests, so an AI-connected session has no pathway to any write-capable endpoint. Fourth, bounded query execution limits: each query is subject to a configurable row-count cap and a server-side timeout, so unintentionally broad queries cannot degrade system availability for other users. The natural-language querying data flow is as follows: the user's question, the MCP tool descriptions (which expose table and column names), and the results returned by each read-only query are passed to the language model, which runs on an external provider's API. These results are pseudonymized, patient-level rows (bounded to at most 500 per query) drawn from the analytical tables; direct identifiers (name, surname, telephone, and e-mail) reside only in the isolated patient_identity table, which is populated for the 46 VRCardio-Explore subjects and is not part of the analytical tables targeted by the querying workflow. The read-only constraint is enforced at the application layer; because it prevents modification but does not by itself restrict which tables may be read, confidentiality currently relies on this table-level isolation and on the deidentified nature of the public sources rather than on a query-time access-control layer, and provisioning a database role scoped to the nonidentifying tables together with a query-time block on the identity table is planned. In transit, all external endpoints use HTTPS/TLS and internal database traffic uses the hosting provider's private encrypted network; raw signal files in object storage are encrypted at rest by the provider. Queries and model responses are logged to an observability service for auditing, and content transmitted to model providers is processed under their respective application programming interface terms; deployments handling identifiable data should restrict requests to providers covered by an appropriate data-processing agreement and use a zero-retention configuration. In the current research deployment, the querying endpoint is not authenticated, and no formal penetration or confidentiality testing has been performed; endpoint authentication and a formal security assessment are required before any deployment involving identifiable data. The read-only design therefore protects data integrity but does not by itself establish confidentiality or General Data Protection Regulation (GDPR) compliance.

Data sources and integration

The platform integrates multiple ECG datasets from prominent public repositories alongside an in-house cohort. Each record is tagged with its original source to ensure full traceability and provenance throughout the analytical workflow. The relational model is centered on the patient entity, linking raw ECG recordings, derived electrophysiological metrics, and per-session anthropometric measurements; derived features are indexed in a dedicated table for high-performance querying of signal-derived parameters.

Natural-language querying workflow

Natural-language queries are processed through a multistep workflow: semantic parsing of the user's research intent; query translation, mapping the natural language to valid SQL/ORM operations; constrained execution, validating the query against the security policies; and structured output generation, presenting the data in a format suitable for clinical interpretation. The interface is model-agnostic: any MCP-compatible LLM client can connect without changes to the database or application layer. In practice, the helper API exposes four options ranked by reasoning capability: Claude Sonnet 4.6 [14] (primary), Gemini 2.5 Pro [15], Gemini 2.0 Flash [15], and Claude Haiku 4.5 [14] (lightweight fallback), with an auto-chain strategy that routes each request to Claude Sonnet 4.6 first and cascades to lighter models on failure or timeout, so querying is not locked to a single provider and can adopt newer models without modifying the server. The exact model identifiers configured at the time of this study were claude-sonnet-4-6 (primary), gemini-2.5-pro and gemini-2.0-flash, and claude-haiku-4-5-20251001 (lightweight fallback). All natural-language querying results reported in this study were produced with claude-sonnet-4-6, the model the platform invokes by default for analytical queries; the remaining models act only as automatic fallbacks on failure or timeout and were not used to generate the reported results. Because commercial model versions and availability change over time, every response returned by the platform records the exact model identifier used, and this identifier is stored with each logged query for traceability.

ECG processing pipeline

The automated ingestion pipeline is executed through five stages: data ingestion from multiple legacy and modern formats; signal standardization to the unified SCP-ECG format [16]; patient-record integration linking signals to existing clinical profiles; metric computation, with automated extraction of heart rate, the clinical intervals (PR, QRS, QT), and heart-rate-variability measures; and storage, with simultaneous archival of structured metadata and raw data.

Statistical analysis

Through the MCP interface and REST API, the platform natively supports descriptive statistics and exploratory analyses, including population-level distributions, cross-source comparisons of signal-derived parameters, and correlations between electrophysiological features and patient-level anthropometric variables, without users writing SQL directly.

Evaluation design

The evaluation is structured around five complementary dimensions: dataset characterization of the integrated repository; semantic accuracy of natural-language querying against a manually curated benchmark with consensus ground-truth SQL; robustness to query phrasing, terminology, and level of detail; illustrative clinical use cases; and an expert clinical assessment comprising a four-task success evaluation by the cardiology team at Hospital Infanta Leonor and a structured usability-and-utility validation in which two senior cardiologists scored their own free-choice research queries.

For the natural-language benchmark, 40 queries were designed following a three-stage blinded protocol. First, the cardiology team independently composed queries representative of real research tasks (cohort selection, diagnostic-code retrieval, statistical summarization) without prior knowledge of the ground-truth SQL. The benchmark covers five clinically motivated categories, with eight queries each: demographic filtering, electrophysiological thresholds, diagnostic-code selection, multicriteria combinations, and metric-distribution requests. Second, two independent annotators (a clinical data scientist and a biomedical informatics researcher) each wrote reference SQL from the natural-language description and published schema alone; interannotator agreement, measured as exact result-set equivalence, was 95% (38 of 40), and the two disagreements were resolved by consensus. Third, each query was submitted verbatim to the MCP-based interface using Claude Sonnet 4.6 [14], which generated SQL solely from the tool descriptions registered on the MCP server; the SQL was executed against the production database, and the resulting cohort was compared with the consensus ground truth. All 40 benchmark queries and all rated cardiologist queries were answered by claude-sonnet-4-6; the autochain fallback models were not invoked, so the reported scores are single-model rather than pooled. Query accuracy was quantified using set-theoretic precision and recall; for aggregate and scalar queries, correctness was defined as an exact numerical match within a tolerance of 0.001. Overall precision and recall are reported with 95% Wilson score confidence intervals (CIs).

For robustness, five query clusters were constructed, each comprising five linguistically distinct reformulations encoding the same clinical intent, varying terminology, syntactic structure, degree of clinical formality, and use of abbreviations. For each cluster, the intersection-over-union (IoU) of the cohorts retrieved across all five phrasings was computed as a pairwise consistency measure. Two senior cardiologists then completed a structured validation protocol assessing usability and utility, rating each response on a five-point Likert scale for medical correctness, whether it answered the question, interpretability, real clinical value, and missing details (reverse-scored), as well as an overall 10-point score.

Reproducibility and Versioning

All reported experiments were run against a single frozen version of the platform and database. The software release used is archived at Zenodo (European Organization for Nuclear Research, Geneva, Switzerland) and GitHub (GitHub, Inc., San Francisco, CA); the natural-language querying model is claude-sonnet-4-6, and the metric-extraction pipeline is the corrected version in which the PR-interval extraction window was widened following the artifact identified during expert evaluation; consequently, all metrics reported in Table 1 and all cohort counts derive from this corrected pipeline. The MCP tool descriptions and the system prompt supplied to the model are reproduced verbatim in the archived repository. Because the PR-interval correction changed the platform during validation, the quantitative benchmark and the representative use cases were re-executed on this final frozen version so that all reported results correspond to the submitted system.

**Table 1:** Summary statistics for signal-derived ECG metrics computed from stored waveforms Computed on n = 385,725 standard 12-lead records; the 46 VRCardio-Explore ECGi recordings and the MIMIC-IV-ECG machine-measured intervals are not included ^†^Excluding records with a degenerate infinite signal-to-noise ratio (signal with zero noise floor) ^‡^QRS duration is obtained by automated signal delineation and is conservative for part of the cohort (particularly the large CODE-15 source) relative to expert annotations; for conduction-based stratification, rely on the SCP-ECG diagnostic codes SD, standard deviation; IQR, interquartile range; QTc, corrected QT interval; SNR, signal-to-noise ratio; ECGi, electrocardiography-imaging; MIMIC-IV-ECG, Medical Information Mart for Intensive Care IV - Electrocardiogram; CODE-15, Clinical Outcomes in Digital Electrocardiology 15

Metric	Mean ± SD	IQR (P25-P75)
Heart rate (bpm)	75.7 ± 22.6	63.2-85.5
QRS duration (ms)^‡^	53.7 ± 24.2	37.5-63.3
QTc Bazett (ms)	357.7 ± 74.0	328.5-382.6
PR interval (ms)	165.8 ± 27.1	148.3-186.2
SNR ratio (dB)^†^	14.1 ± 10.7	4.8-22.3

Regarding the dates, the natural-language querying benchmark was executed with claude-sonnet-4-6 in August 2026, and the expert clinical assessment was conducted in June 2026.

## Results

Dataset characterization

The platform currently integrates data from nine sources, encompassing approximately 1,185,800 ECG records from approximately 432,000 distinct patients (Table 2). The dominant contributor by volume is MIMIC-IV-ECG [3], derived from the MIMIC-IV database [17], which accounts for approximately 800,000 records; as the corresponding raw binary files are not redistributed under the dataset's data use agreement, these records have no stored signal files, and their interval metrics are ingested from the machine measurements rather than computed from the waveform (signal-derived metrics such as SNR and heart-rate variability are therefore unavailable). The remaining approximately 385,000 records, drawn from eight additional sources, are stored with their raw signal files. The seven standard 12-lead sources (385,725 records) have fully computed electrophysiological metrics; the 46 VRCardio-Explore recordings, acquired with a 34-electrode ECGi montage rather than a 12-lead configuration, retain anthropometric and raw-signal data but do not carry the computed 12-lead interval metrics. Every record carries a metrics row, populated with computed values (the seven standard 12-lead sources) or ingested machine measurements (MIMIC-IV-ECG); the VRCardio-Explore rows remain unpopulated for the 12-lead interval fields.

**Table 2:** Per-source composition of the VRCardio-Helper-Database "Ingested" indicates that interval metrics were taken from the source machine measurements rather than computed from the waveform MIMIC-IV-ECG, Medical Information Mart for Intensive Care IV - Electrocardiogram; CODE-15, Clinical Outcomes in Digital Electrocardiology 15; PTB-XL, Physikalisch-Technische Bundesanstalt XL; CPSC 2018, China Physiological Signal Challenge 2018; LUDB, Lobachevsky University Electrocardiography Database; SCP-ECG, Standard Communications Protocol for Computerized Electrocardiography; ECG, electrocardiography; ECGi, electrocardiography-imaging; PII, personally identifiable information

Source	ECG records	Signal file	Metrics	Notes
MIMIC-IV-ECG [3]	800,035	No	Ingested	Largest source; raw files not redistributed; interval metrics ingested from source machine measurements
CODE-15 [18]	345,644	Yes	Yes	Brazilian national cohort, 400 Hz
PTB-XL [1]	21,778	Yes	Yes	12-lead, 500 Hz, SCP-ECG codes
Shaoxing-Chapman [11]	10,646	Yes	Yes	12-lead, 500 Hz, rhythm labels
CPSC 2018 [19]	6,877	Yes	Yes	Chinese Cardiovascular Society challenge
ISP [20,21]	475	Yes	Yes	12-lead ECGs with expert P/QRS/T delineation annotations
LUDB [22]	200	Yes	Yes	Lobachevsky University, fully delineated
QT database [23]	105	Yes	Yes	PhysioNet, annotated QT boundaries
VRCardio-Explore [24,25]	46	Yes	No	Pilot in-house cohort (CE-certified Class IIb device; patent-protected); ECGi via 34-electrode textile data-acquisition system, so 12-lead interval metrics are not computed; includes PII and anthropometrics
Total	1,185,806	-	-	-

The VRCardio-Explore cohort, the smallest source by record count (46 recordings), comprises cases selected from clinical data collected under the EU-funded VR-CARDIO project [24], coordinated by Spika Tech SL (Spain) under the European Innovation Council. The VR-CARDIO medical device software developed by Spika Tech SL in the parent project achieved CE certification as a Class IIb medical device (certificate C08 ES044822 2512, issued on December 1, 2025, by the notified body HTCert (Nicosia, Cyprus), identification number 2803, and valid until November 10, 2030). This certification covers the VR-CARDIO electrical-anatomical reconstruction and three-dimensional visualization software and not the research database and natural-language querying platform described in this article, which is not itself a certified medical device and is intended for research and cohort-querying use only. The underlying electrocardiographic technology is protected by European patent EP23382800 [25]. Although small (reflecting the stringent clinical selection criteria of a prospective real-world validation study), it is the only source providing simultaneously acquired anthropometric measurements, pseudonymized identity records, and raw ECGi signals (from a textile data-acquisition system bearing 34 dry electrodes, from which non-invasive electro-anatomical maps are reconstructed, rather than a standard 12-lead montage) within the same recording session, enabling forms of multimodal analysis not feasible with any of the public datasets.

Missingness of Key Variables

Of the 432,338 patients, sex was male for 114,524 (26.5%) and female for 156,461 (36.2%), and was unspecified for 161,353 (37.3%). A valid age was available for 270,628 patients (62.6%), with a mean of 52.1 ± 19.8 years; age was missing or noninformative for the remaining 161,710 (37.4%). Ages outside the plausible human range, defined here as 0 or below or above 120 years, are treated as implausible; the only such value present is 300, a placeholder used in the public PTB-XL release to encode patients older than 89 (censored age), which affects 257 patients (293 records), all from PTB-XL, and which is excluded from age summary statistics and from analyses that use age as a covariate while the records are retained in the database and remain retrievable by queries. Ethnicity is not populated for the large public sources and is therefore absent for most of the repository. Computed ECG metrics (heart rate, PR, QRS, QTc, SNR, and the arrhythmia flag) are available for 347,296 records, whereas MIMIC-IV-ECG (800,035 records) is integrated without computed metrics by design, and SCP-ECG diagnostic codes are present only for the four annotated sources (PTB-XL, Shaoxing-Chapman, China Physiological Signal Challenge 2018 (CPSC 2018), and Clinical Outcomes in Digital Electrocardiology (CODE)-15). Missingness is strongly source-dependent, and a per-source breakdown is reported below.

Sex Missingness by Source

Missing sex is not randomly distributed but concentrated in a single source. Sex is recorded for essentially all patients in CODE-15, PTB-XL, Shaoxing-Chapman, CPSC 2018, ISP, and Lobachevsky University Electrocardiography Database (LUDB) (each below 0.1% missing) and for all but one patient in the VRCardio-Explore cohort; the MIMIC-IV-ECG subset does not release patient sex and accounts for the large majority of the 161,353 patients with unspecified sex, with the small QT Database (90 of 105 patients) contributing the remainder. The high overall unspecified-sex proportion (37.3%) therefore reflects the integration of one large source that does not distribute sex rather than within-source data loss, and sex-stratified analyses are effectively restricted to the non-MIMIC subset.

Signal-derived metrics were computed from the raw waveform for the 385,725 standard 12-lead records with stored signal files (the 46 VRCardio-Explore ECGi recordings are excluded); Table 1 summarizes their distribution. The mean heart rate of 75.7 ± 22.6 bpm and the mean PR interval of 165.8 ± 27.1 ms fall within the expected adult ranges. The mean QRS duration of 53.7 ± 24.2 ms lies below the normal adult range (approximately 80-100 ms): although the automated delineation is accurate for the standard 12-lead sources (for example, 94.5 and 90.8 ms for PTB-XL and Shaoxing-Chapman, respectively), the pooled mean is dominated by the large CODE-15 cohort, for which the QRS-offset detection is conservative; the numeric QRS column should therefore be cross-checked against the SCP-ECG diagnostic codes for conduction-based stratification. The mean QTc (Bazett) is 357.7 ± 74.0 ms, and the mean SNR, computed after excluding records with a degenerate infinite value arising from artificially noise-free signals, is 14.1 ± 10.7 dB. Among the sources carrying structured diagnostic annotations, the prevalence of arrhythmia diagnoses (SCP-ECG rhythm-disorder codes) ranges from 2.0% in CODE-15 to 27.8% in the Shaoxing-Chapman dataset (Table 3), consistent with the different selection criteria and patient populations of each repository. More broadly, these signal-derived metrics are produced by an automated extraction pipeline, and their measurement accuracy is distinct from the accuracy with which a natural-language query is translated into the intended database operation; apart from QRS duration, which was quantified against the expert wave delineations of the LUDB dataset (Discussion), the derived PR, QT, and QTc intervals, heart rate, and heart-rate-variability measures have not been independently validated against expert annotations or source measurements and should be validated before being used for clinical phenotype identification.

**Table 3:** Prevalence of arrhythmia diagnoses among sources with structured diagnostic annotations Datasets that provide waveform delineations only and no diagnostic labels (ISP, LUDB, QT Database), together with MIMIC-IV-ECG and VRCardio-Explore, are omitted. Arrhythmia is defined as the presence of at least one SCP-ECG rhythm-disorder code (atrial fibrillation or flutter, supraventricular and ventricular tachycardias, atrial or ventricular ectopy, paced rhythm, bigeminy/trigeminy), excluding sinus bradycardia, sinus tachycardia, and normal sinus rhythm dx, diagnoses; CPSC 2018, China Physiological Signal Challenge 2018; PTB-XL, Physikalisch-Technische Bundesanstalt XL; CODE-15, Clinical Outcomes in Digital Electrocardiology 15; LUDB, Lobachevsky University Electrocardiography Database; MIMIC-IV-ECG, Medical Information Mart for Intensive Care IV - Electrocardiogram; SCP-ECG, Standard Communications Protocol for Computerized Electrocardiography

Source	Total	Arrhythmia dx	Prevalence (%)
Shaoxing-Chapman [11]	10,646	2,964	27.8
CPSC 2018 [19]	6,877	1,833	26.7
PTB-XL [1]	21,778	1,470	6.7
CODE-15 [18]	345,644	7,033	2.0

The most frequently annotated SCP-ECG diagnostic code across all annotated sources is normal sinus rhythm, appearing in 144,488 records, followed by right bundle branch block (11,524), sinus bradycardia (SB; 9,492), sinus tachycardia (9,148), atrial fibrillation (AF; 7,470), first-degree atrioventricular block (6,434), and left bundle branch block (6,260). The co-occurrence of both AF and AFIB codes in the frequency distribution reflects the use of different annotation schemas across the source datasets (for example, CODE-15 uses AF, whereas the Shaoxing-Chapman and CPSC datasets use AFIB).

Natural-language query evaluation

Query interpretation achieved an overall precision of 90% (95% CI, 77-96) and recall of 88% (95% CI, 75-95) across the 40-query benchmark (Table 4). Demographic filtering, multicriteria combinations, and metric distributions were answered essentially perfectly; the electrophysiological-threshold and diagnostic-code categories accounted for the reduced aggregate figures. A query was considered fully correct if both precision and recall equaled 1.0; partially correct if it retrieved a nonempty cohort that overlapped but did not exactly match the reference (with precision or recall below 1.0); and failed otherwise.

**Table 4:** Natural-language query evaluation results N = 40 queries, eight per category; values are mean ± standard deviation over queries within each category; overall figures are given with 95% Wilson score confidence intervals in parentheses HR, heart rate; QRS, QRS duration; QTc, corrected QT interval; PR, PR interval; SNR, signal-to-noise ratio; SCP-ECG, standard communication protocol for computer-assisted electrocardiography

Category	Description	Precision (%)	Recall (%)
Demographic filtering	Age, sex, source constraints	100 ± 0	100 ± 0
Electrophysiological thresholds	HR, QRS, QTc, PR, SNR constraints	81 ± 35	88 ± 33
Diagnostic code selection	SCP-ECG annotation filtering	68 ± 41	100 ± 0
Multicriteria combinations	Two or more simultaneous conditions	100 ± 0	100 ± 0
Metric distributions	Aggregates, percentiles, stratified statistics	100 ± 0	100 ± 0
Overall		90	98

Inspection of the seven failing or partially failing queries revealed three distinct error modes, each attributable to a mismatch between the model's general knowledge and the specific representational conventions of this schema rather than to a fundamental limitation of the underlying model. A per-query error analysis of the live run identified two error modes, both associated with identifiable schema and terminology mismatches rather than demonstrably with limitations of the model. First, metric unit conventions: the pr_interval, qrs_duration, and qtc_bazett columns store values in seconds, so clinically natural millisecond phrasings were sometimes applied without conversion. For "QRS duration between 60 and 100 ms," the model queried BETWEEN 60 AND 100 in seconds and retrieved no records (a failed query), and for "corrected QT interval below 360 ms," it queried <360 rather than <0.360, matching essentially the entire table and reducing precision to 0.44; the conversion was applied correctly for other interval queries (for example, PR interval greater than 120 ms). Adding explicit unit annotations to each metric column in the MCP tool description may reduce or eliminate this ambiguity. Second, overinclusive diagnostic-code selection: for several code-based queries, the model supplemented the SCP-ECG code filter with free-text scp_description ILIKE patterns and additional synonyms which, compounded by operator precedence in the generated OR clauses, retrieved cohorts substantially larger than the consensus reference (for example, AF, 88,947 generated vs. 10,516 reference; SB, 106,438 vs. 9,492). Recall was preserved because the true cohort remained a subset, but precision fell. Constraining the tools to the curated SCP-ECG code vocabulary and discouraging free-text description matching may reduce or eliminate these over-retrievals. These are observed, potentially correctable error modes; establishing that they arise from schema documentation rather than from model limitations would require correcting them and rerunning the benchmark, an ablation we have not performed.

Robustness analysis

Cohort retrieval was fully consistent (mean pairwise IoU 1.00) for the demographic, electrophysiological-threshold, multicriteria, and mixed-clinical clusters, and 0.86 for the diagnostic-code cluster (where AF/AFIB synonymy reduced overlap), giving an overall mean IoU of 0.97. The complete set of query reformulations for each cluster, with the per-cluster IoU, is listed in the Appendix.

Clinical use cases

The following use cases illustrate representative analytical workflows executed through the natural-language querying interface, demonstrating the system's ability to support hypothesis generation and exploratory research without requiring knowledge of the underlying schema. All queries in this section were submitted through the web-based chat interface (Figure 2).

**Figure 2:**
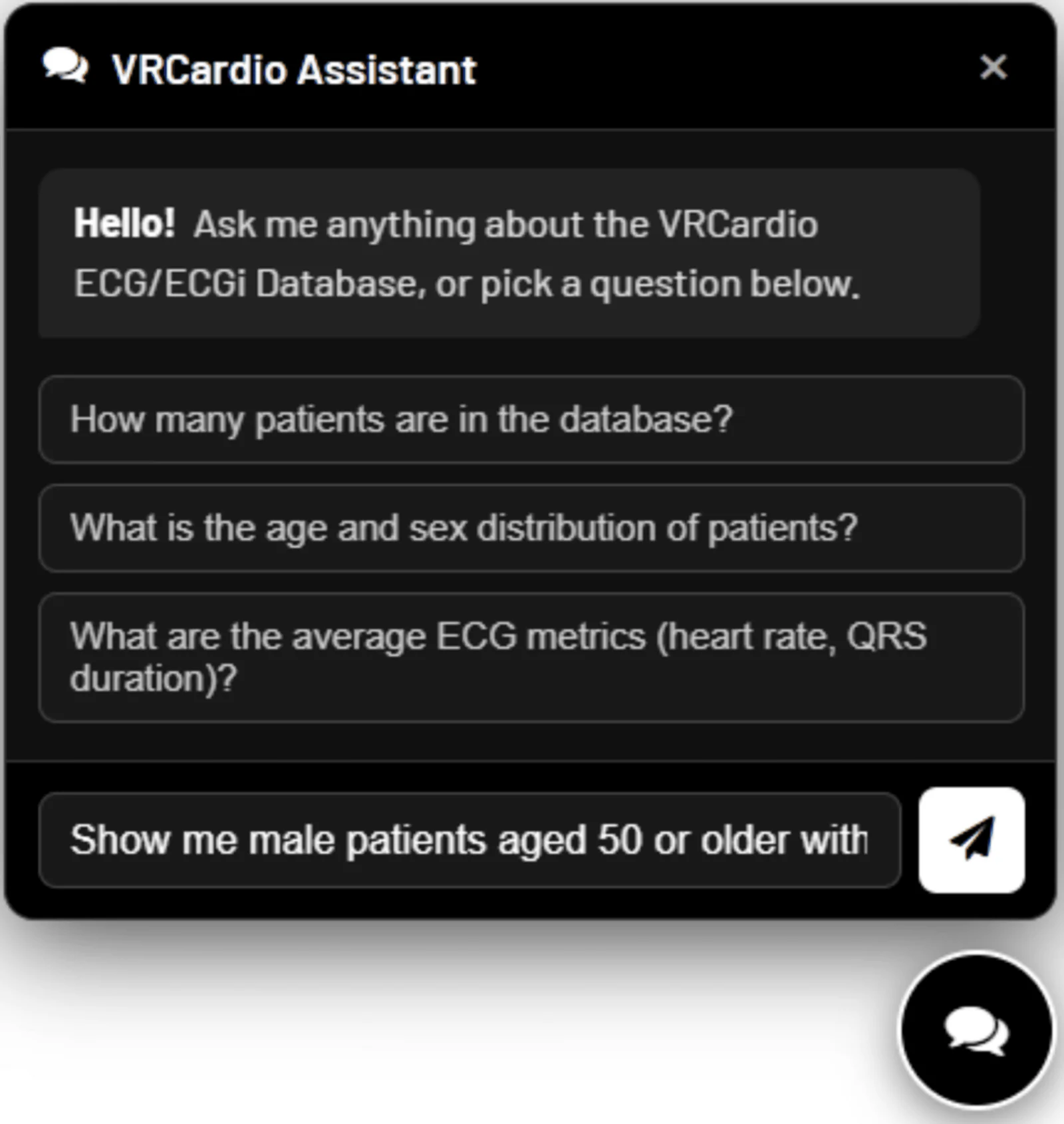
Web-based natural-language query interface of the VRCardio-Helper-Database The user types a clinical research question in plain language; the system translates it into a structured database query, executes it against the production database, and returns the results in a readable format, without requiring the user to write SQL SQL, structured query language; ECG, electrocardiography; ECGi, electrocardiographic-imaging

Use case 1, cross-source cohort selection for elderly men with AF and prolonged QTc: the query "Show me male patients aged 50 or older with AF and a corrected QT interval above 450 milliseconds" was translated into a join across patients, ecg_records, and ecg_metrics, filtering on sex, age, SCP code, and qtc_bazett > 0.450. It returned 1,034 ECG records from four sources (PTB-XL, Shaoxing-Chapman, CODE-15, CPSC 2018), corresponding to 876 distinct patients, with QTc from 450 to >700 ms and ages spanning 50-92 years, surfacing rare candidate phenotypes across heterogeneous datasets in a single interaction. Because a patient may contribute several recordings, this result is reported at the ECG-record level; patient-level questions are answered by selecting distinct patient identifiers, and record-level counts, which can overweight patients with repeated recordings, are distinguished from patient-level counts throughout. The most extreme values (above 700 ms) warrant caution, since Bazett's correction overestimates QTc at high heart rates and should be confirmed with a rate-robust correction (for example, Fridericia).

Use case 2, sex differences in ventricular conduction: the query "Compare QRS duration between men and women across all sources except MIMIC" returned mean QRS durations of 50.6 ± 21.9 ms over female records (n = 225,191) and 56.3 ± 25.6 ms over male records (n = 160,533), with medians of 41.9 and 45.3 ms. The direction (longer QRS in men) is consistent with the established physiological sex difference [26,27]; the absolute values are affected by the interval-extraction behavior noted for Table 1. Because this comparison relies directly on the numeric QRS column, the substitution of SCP-ECG diagnostic codes recommended elsewhere for conduction analyses does not apply here; accordingly, only the direction of the sex difference should be interpreted, and the absolute QRS values will require a validated delineation model before they can be used quantitatively.

Use case 3, age-related heart-rate trends: the query "What is the average heart rate across different age groups, excluding MIMIC?" stratified patients into four age groups, revealing a monotonically increasing trend: 73.1 ± 16.6 bpm under 30 years (n = 57,212), 74.7 ± 18.4 bpm at 30-49 years (n = 100,218), 75.7 ± 22.9 bpm at 50-69 years (n = 122,176), and 79.9 ± 25.9 bpm at 70 years or older (n = 100,932), a direction compatible with documented age-related autonomic changes [28]. This is, however, an unadjusted comparison across heterogeneous datasets, and the trend could also reflect differences in comorbidities, medications, referral patterns, source populations, or repeated ECG measurements per patient; the widening standard deviation with age is a descriptive observation and does not, by itself, establish greater heterogeneity in autonomic regulation. A formal adjusted analysis would be required to attribute the trend to autonomic mechanisms.

Use case 4, multimodal exploration in the VRCardio-Explore cohort: capitalizing on the cohort's multimodal structure, the query "For VRCardio-Explore patients with height and weight recorded, show their body mass index together with the associated ECG recording" joined patient_measurements, ecg_records, and patients, returning body mass index values from 21.8 to 30.7 kg/m^2^ linked to each patient's simultaneously acquired session. Because VRCardio-Explore uses a 34-electrode ECGi montage, the 12-lead interval metrics are not derived; the cohort's distinctive value is the joint availability of anthropometric measurements and raw ECGi signals within the same session, a combination not feasible with any single existing public dataset.

Expert clinical assessment

To assess practical utility, the cardiology team at Hospital Infanta Leonor executed the four use-case tasks through the web-based chat interface, without prior knowledge of the schema or SQL, and independently reviewed the results for clinical plausibility against established electrophysiological reference values and their clinical judgment. The team completed all four tasks without external assistance, and in every case, the relative and population-level findings were clinically plausible and consistent with known physiology; for Tasks 1 and 2, this refers to the aggregate results, as the absolute interval values depend on metrics affected by the extraction artifact discussed in the Discussion section (Table 5). The evaluators noted that querying multiple heterogeneous datasets in natural language and obtaining structured results in seconds substantially lowered the technical barrier usually encountered with ECG repositories and would accelerate hypothesis generation.

**Table 5:** Expert clinical assessment: task-success summary (cardiology team, Hospital Infanta Leonor) All tasks were completed without SQL assistance. "Clinically plausible" refers to the relative/aggregate finding; the absolute interval values underlying Tasks 1 and 2 are subject to the metric-extraction artifact discussed in the Discussion section AF, atrial fibrillation; QTc, corrected QT interval; QRS, QRS duration; BMI, body mass index; PTB-XL, Physikalisch-Technische Bundesanstalt XL; CODE-15, Clinical Outcomes in Digital Electrocardiology 15; SQL, structured query language

Task	Clinical intent	Completed	Clinically plausible	Datasets spanned
1	Cross-source cohort: males ≥50 years, AF, QTc >450 ms	Yes	Yes	PTB-XL, Shaoxing-Chapman, CODE-15 [1,11,18]
2	Sex differences in QRS duration	Yes	Yes	Seven 12-lead sources [1,11,18-23]
3	Age-stratified mean heart rate	Yes	Yes	Seven 12-lead sources [1,11,18-23]
4	Multimodal BMI and recording-session retrieval	Yes	Yes	VRCardio-Explore [24,25]
Overall		4/4	4/4	-

Beyond this plausibility check, two senior cardiologists (both coauthors of this study, from the Cardiology Department of Hospital Infanta Leonor) independently completed a structured validation protocol. They posed 35 queries in total, of which 28 returned a response, a response-completion rate of 80%; the remaining seven (a 20% no-response rate) encountered transient service-availability timeouts at the hosted-assistant layer. The aggregate ratings below are computed only over the 28 responded queries and therefore represent an upper bound on real-world usability. As a sensitivity analysis that treats the seven timeouts as failures (scored 0 of 10), the completion-adjusted mean overall quality falls from 7.6 to approximately 6.1 of 10. Across the responses that returned content, the interface scored highly on the dimensions tied to clinical trust: mean medical correctness 4.2/5 and interpretability 4.6/5, with 20 of 28 responses rated at least 8/10 (13 at least 9/10) and several analyses rated maximally useful (10/10) (Table 6). The cardiologists particularly valued the clinically coherent interpretation beyond the literal query, the transparent flagging of data provenance and privacy constraints, and a self-critical meta-analysis that correctly diagnosed a measurement convention in the stored PR-interval metric. One evaluator, discussing a proposed age threshold for atrial-fibrillation screening, noted that the most recent (2024) European Society of Cardiology guidelines are also available [29]. Clinical value was scored more conservatively (mean 3.0/5), as some correct answers merely restated established knowledge; the main usability issue was intermittent service availability timeouts during complex multi-criteria queries.

**Table 6:** Aggregate cardiologist ratings from the structured validation protocol Computed over the responses that returned content (excluding transient service-availability timeouts, for which no answer was produced). Likert dimensions are scored 1-5 (higher is better, except missing details, which is reverse-scored so lower is better); overall quality is scored 1-10 The downward arrow (↓) marks the reverse-scored dimension (missing important details), for which a lower score is better n, number of rated responses; Med. correct, medically correct; AQ, answers the question; Clin. value, real clinical value; Missing det., missing important details

Evaluator	Med. correct	AQ	Interpretable	Clin. value	Missing det. ↓	Overall (/10)
Cardiologist A (n = 15)	3.8	4.2	4.5	3.2	2.4	6.9
Cardiologist B (n = 13)	4.8	4.9	4.8	2.9	1.5	8.4
Combined (n = 28)	4.2	4.5	4.6	3.0	2.0	7.6

## Discussion

The clinical data platform presented in this study addresses a persistent gap in cardiovascular research: the limited availability of unified, scalable, and multimodal infrastructures for ECG-centric analysis. By integrating raw electrophysiological signals with patient-level phenotypic data, the proposed system supports cohort definition and exploratory analysis that extend beyond the constraints of traditional dataset-centric studies.

Integration and multimodality

A key strength is the relational data model, which explicitly links ECG-derived metrics with anthropometric and demographic variables. In contrast to repositories such as PhysioNet [30], where signal data and clinical context are often partially decoupled, this approach enables joint exploration of electrophysiological and phenotypic dimensions relevant to personalized cardiology, where individual variability may influence ECG morphology and clinical outcomes.

Standardization and reproducibility

The end-to-end ingestion and processing pipeline improves reproducibility, a known challenge in large-scale ECG research: standardizing heterogeneous formats into a unified representation and systematically computing signal-derived metrics reduces dataset-specific preprocessing variability, while integrating multiple public and proprietary datasets increases population diversity. However, differences in acquisition protocols, selection criteria, annotation standards, and missingness across sources may also introduce substantial source-specific bias, so greater diversity does not, by itself, guarantee the generalizability of downstream analyses.

Natural-language data access

The AI-assisted querying interface lets users interact with the database in natural language: cohort definition, which conventionally requires familiarity with database schemas and SQL, is expressed in clinically meaningful terms. Because language understanding is delegated to a multilingual LLM, the interface can, in principle, accept queries in any language the model supports, which may reduce linguistic barriers for non-English-speaking users without additional localization effort. This multilingual capability is a design property of the underlying model and was not experimentally evaluated in this study.

This database-level approach is architecturally distinct from signal-level ECG question-answering systems such as ECG-QA [8] and Q-HEART [9], which train specialized encoders to answer questions about a single waveform (what does this ECG show?) and are evaluated on PTB-XL alone. The present system instead answers population-level queries (which patients meet these criteria?) across nine heterogeneous sources with no ECG-specific model training, making it immediately extensible. For context, frontier models on the BiomedSQL benchmark [10] reach 58%-62% execution accuracy without domain adaptation, whereas the present system reaches 90% precision and 88% recall on a production-deployed clinical database. These figures are not directly comparable, because BiomedSQL and the present benchmark differ in their datasets, query distributions, task complexity, and performance metrics; the comparison therefore illustrates the benefit of curated tool design and domain-adapted schema annotation in a specialized setting rather than establishing superiority over prior systems. These metrics quantify query-translation fidelity, that is, whether a natural-language request is mapped to the intended database operation, and are conceptually distinct from the accuracy of the ECG-derived measurements themselves: a query can retrieve exactly the intended cohort while the underlying metric on which it filters (for example, QRS duration) carries its own extraction error, as the markedly underestimated pooled QRS duration illustrates.

The backbone LLM influences querying performance. Claude Sonnet 4.6 [14] was selected here for its instruction-following and tool-use capabilities, central to the MCP paradigm; the model-agnostic architecture lets performance across families (for example, vs. Gemini 2.5 Pro [15]) be compared without changing the server, providing a tractable path for ongoing benchmarking. The error modes identified above (unit ambiguity, dataset-specific code naming, cross-source synonyms) were each associated with identifiable schema and terminology mismatches. Establishing that they stem from schema documentation rather than from model limitations would require correcting these mismatches in the MCP tool descriptions and rerunning the benchmark, an ablation we have not yet performed; if confirmed, such corrections would be expected to benefit all supported models.

The system also enforces safety constraints (read-only execution, bounded query scope) to control interaction with sensitive data; while accuracy is high, further validation is required in more complex and ambiguous clinical scenarios.

Architectural considerations and clinical applicability

The platform supports both retrospective analysis and prospective clinical workflows, and the separation of PII from analytical data is designed to support pseudonymized access aligned with frameworks such as the General Data Protection Regulation, although, as detailed in the Materials and Methods section, the read-only design protects data integrity but does not by itself establish confidentiality or GDPR compliance, which would require the additional access-control, authentication, retention, and security-testing measures described there; scalable backend technologies handle large volumes of ECG data. The platform is intended primarily as a research and cohort-querying tool for hypothesis generation rather than as a clinical decision-support system: combining signal-derived features with patient-level variables and querying them intuitively may accelerate exploratory research, but direct impact on clinical decision-making has not been evaluated and would require separate prospective validation.

Limitations and future directions

Several limitations should be acknowledged. First, integrating heterogeneous datasets may introduce biases from differences in acquisition protocols, populations, and annotation standards. Second, anthropometric data are available only for a subset of the cohort, constraining certain multimodal analyses. Third, the querying interface requires further evaluation in real-world settings, particularly for ambiguous or underspecified queries. The structured clinical validation reported above and in the Appendix surfaced two concrete avenues for improvement. The first was intermittent service-availability timeouts on a subset of the more complex multicriteria queries, a concurrency and request-timeout issue at the hosted-assistant layer rather than a semantic limitation, addressable through queue management and retry handling. The second concerned the automated interval-extraction stage, which the evaluation helped characterize. An initial PR-interval truncation near 0.150 s, noticed during evaluation and correctly diagnosed by the interface itself, was traced to an overly narrow extraction window; widening the window and recomputing the affected columns resolved it, and the corrected PR distribution now falls within the physiological range (Table 2). For QRS duration, the automated delineation is accurate on the standard 12-lead sources (for example, 94.5 and 90.8 ms for PTB-XL and Shaoxing-Chapman) but conservative on part of the pooled cohort: quantified against the expert wave delineations of the LUDB dataset, it returns a lower mean QRS duration (approximately 46 vs. 103 ms), a bounded and well-understood delineation limit rather than a physiological finding. This does not affect the platform's querying capability or its diagnostic-code-based analyses; a validated delineation model (for example, a trained delineator such as that of Avetisyan et al. [21]) will replace the current heuristic in future work, and in the interim, conduction-based analyses use the SCP-ECG diagnostic codes rather than the numeric QRS values (Table 2). The QT and QTc estimates have not been independently validated against expert measurements or source annotations; the low pooled QTc distribution and the extreme values observed in Use Case 1 indicate that the automated T-wave offset detection is unreliable, particularly at the distribution tails. The QT and QTc columns should therefore be treated as relative, screening-level indicators rather than validated measurements and should not be used to identify high-risk clinical phenotypes, such as long-QT candidates, without independent validation; the planned validated delineation model will address QT-offset detection alongside QRS. Fourth, the expert clinical assessment was conducted with two cardiologists who are also coauthors of this work and are based at a single institution; their involvement and the single-center panel may introduce evaluator bias and limit the generalizability of the ratings, so it should be read as an internal expert evaluation rather than independent validation. Broader conclusions about usability require a formal multiuser study with independent clinicians from additional centers, which is planned as immediate future work. Fifth, the quantitative benchmarks are of modest size (40 natural-language queries; five robustness clusters of five reformulations each; 28 rated expert responses), so the reported precision, recall, and intersection-over-union carry correspondingly wide CIs; we therefore present them as an initial, carefully curated evaluation, with a larger, multicenter benchmark planned. In addition, the quantitative benchmark relied on a single primary model (Claude Sonnet 4.6), so results may not transfer across model families; the reliance on heterogeneous public datasets may introduce dataset-selection bias arising from differing acquisition and annotation practices; the high proportion of unspecified sex (37.3% of patients) constrains sex-stratified analyses, which apply only to the subset with recorded sex rather than to the full repository; and the security safeguards, while enforced by design (read-only execution, bounded queries, and PII isolation), have not undergone formal security or penetration testing. Sixth, the analyses depend on automatically extracted ECG measurements whose errors have direct interpretive implications: the underestimated QRS duration and the unreliable QT/QTc offset detection can bias metric-based cohort definitions and prevalence estimates and, as noted above, should not be used for high-risk phenotype identification without independent validation. Reference intervals are also age-dependent, and the expert evaluation confirmed that applying adult ECG-normality criteria to pediatric recordings (for example, the 0-9-year age group) yields misleading results; therefore, pediatric and neonatal ECGs require age-specific interpretation not provided by the adult thresholds used in the current metrics. In addition, the platform has not undergone external or prospective validation on independent cohorts, which remains necessary before any clinical application.

Future work will expand the platform's multimodal scope (imaging modalities, reconstructed cardiac maps, and longitudinal electronic health records), run a prospective usability study with independent cardiologists using standardized instruments such as the System Usability Scale, and integrate predictive models within the querying framework for clinical decision support.

## Conclusions

We present VRCardio-Helper-Database, a unified, multimodal platform for ECG-centered data integration and exploration, augmented by an artificial-intelligence-assisted natural-language interface that enables intuitive access to complex clinical datasets. The platform integrates approximately 1,185,800 ECG records from approximately 432,000 patients across nine sources and links electrophysiological signals to demographic and anthropometric data, enabling clinicians to define cohorts and run population-level analyses in plain language without writing SQL.

Across a 40-query benchmark, the interface reached 90% precision and 88% recall, and two senior cardiologists rated its responses highly for medical correctness and interpretability; the few errors were associated with identifiable schema and terminology mismatches and are therefore likely addressable at the level of the schema documentation rather than through model retraining. By reducing the programming expertise required to work with multimodal ECG data and by exposing electrophysiological and phenotypic variables together, the platform offers a scalable, reproducible foundation for hypothesis generation in cardiovascular research. The detailed multimodal layer, comprising simultaneously acquired anthropometric and ECGi measurements, is currently confined to the 46-record VRCardio-Explore cohort, whereas the remainder of the repository (approximately 1.19 million ECG records) comprises ECG signals with associated metadata and derived metrics; extending this multimodal layer to a larger cohort is a priority for future work. Prospective, multicenter validation and an expanded multimodal scope are the immediate next steps.

## Figures and Tables

**Figure 3:**
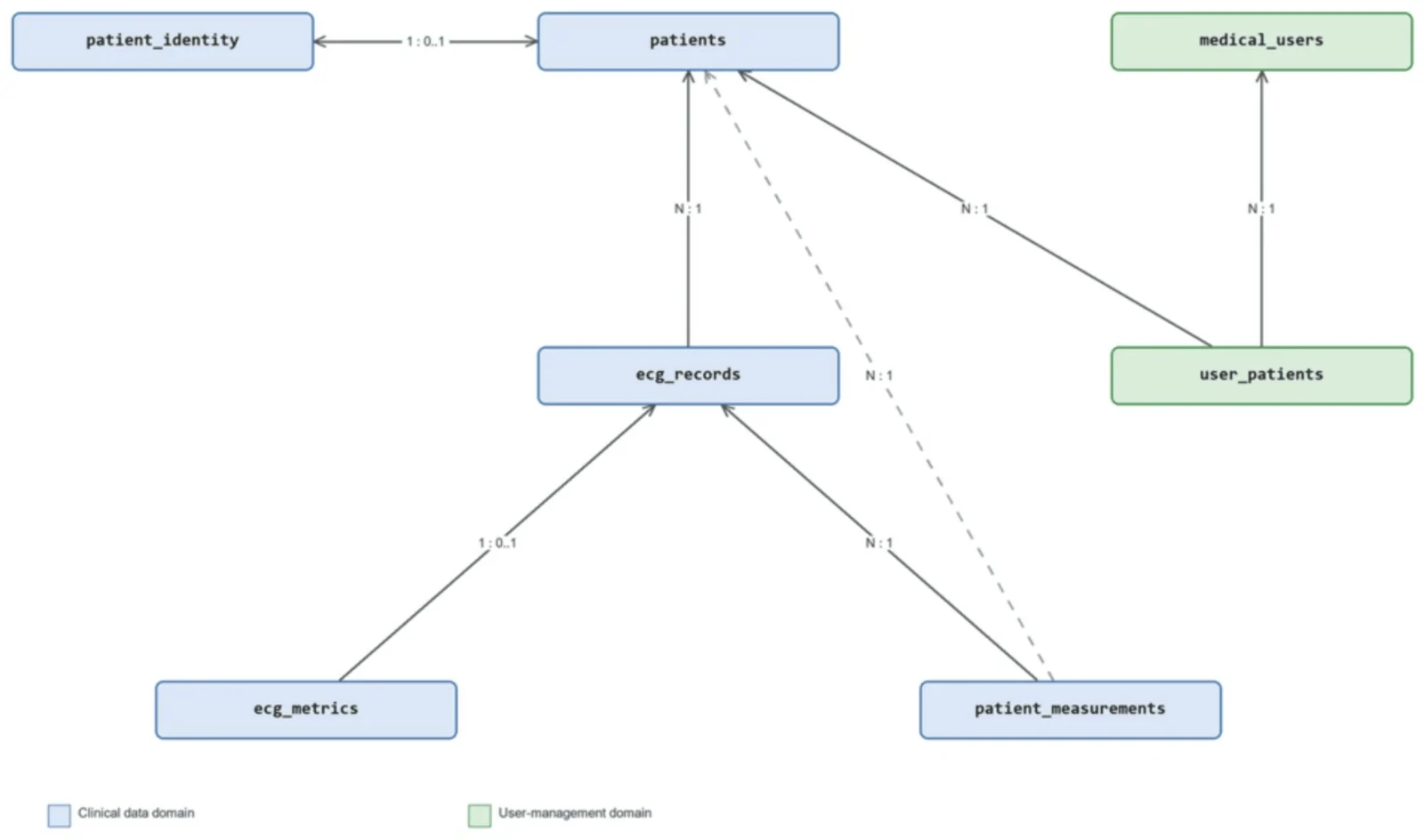
Entity-relationship diagram of the VRCardio-Helper-Database schema The schema comprises seven tables across two domains: a clinical data domain (blue) and a user-management domain (green). Solid arrows indicate mandatory foreign-key relationships; the dashed arrow denotes a secondary foreign key. Cardinality follows child:parent notation (for example, N:1 means many child records reference one parent) Image credit: This figure was created by the author Carlos Rodrigo Rivero using the Python Pillow (Python Imaging Library) imaging library (Jeffrey “Alex” Clark and contributors; open-source software)

## Appendices

Detailed system architecture

The complete entity-relationship diagram of the VRCardio-Helper-Database schema, including all seven tables, primary keys, and foreign-key relationships, is shown; tables are color-coded by functional domain (clinical data versus user management).

The complete column-level schema

**Table 7:** Column-level schema of the VRCardio-Helper-Database All seven tables are listed with their PostgreSQL column types and roles PK, primary key; FK, foreign key; UUID, universally unique identifier; NN, normal-to-normal interval; ECG, electrocardiography; MIMIC-IV-ECG, Medical Information Mart for Intensive Care IV - Electrocardiogram; SCP-ECG, Standard Communications Protocol for Computerized Electrocardiography; QRS, QRS duration; QTc, corrected QT interval

Table	Column	Type	Description
patients	patient_id	UUID (PK)	Internal surrogate key
	db_patient_id	VARCHAR(255)	Original dataset-specific patient identifier
	sex	VARCHAR(20)	Biological sex (M, F, or NULL)
	age	INTEGER	Age in years at recording time
	ethnicity	VARCHAR(100)	Ethnicity (where available)
patient_identity	patient_identity_id	UUID (PK)	Internal surrogate key
	patient_id	UUID (FK, UNIQUE)	References patients; UNIQUE enforces 1:1
	name	VARCHAR(255)	Given name
	surname	VARCHAR(255)	Family name
	birth_date	DATE	Date of birth
	phone	VARCHAR(50)	Contact phone number
	email	VARCHAR(255)	Contact email address
ecg_records	ecg_id	UUID (PK)	Internal surrogate key
	db_ecg_id	VARCHAR(255)	Original dataset-specific recording identifier
	patient_id	UUID (FK)	References patients.patient_id
	uploaded_by_user_id	UUID (FK)	References medical_users (nullable; set on clinical uploads)
	source	VARCHAR(50)	Dataset provenance tag (e.g., ptbxl, mimic)
	recording_date	TIMESTAMP	Date and time of ECG acquisition
	n_leads	INTEGER	Number of leads (typically 12)
	sample_rate_hz	INTEGER	Sampling frequency (Hz)
	duration_seconds	FLOAT	Recording duration in seconds
	device	VARCHAR(255)	Acquisition device model (nullable)
	bandwidth	VARCHAR(100)	Signal bandwidth specification (nullable)
	filtering	VARCHAR(255)	Applied signal filtering description (nullable)
	filename	VARCHAR(1024)	Cloudflare R2 object key (NULL for MIMIC-IV-ECG)
	scp_codes	VARCHAR[]	Array of SCP-ECG diagnostic codes
	scp_description	TEXT	Free-text SCP annotation description (nullable)
ecg_metrics	ecg_id	UUID (PK/FK)	Shared PK; references ecg_records.ecg_id
	heart_rate_bpm	FLOAT	Mean heart rate (bpm)
	rr_interval_avg	FLOAT	Mean RR interval (seconds)
	pr_interval	FLOAT	PR interval duration (seconds)
	qrs_duration	FLOAT	QRS complex duration (seconds)
	qt_interval	FLOAT	QT interval duration (seconds)
	qtc_bazett	FLOAT	Bazett-corrected QTc (seconds)
	heart_axis	FLOAT	Cardiac electrical axis (degrees)
	p_wave_amplitude	FLOAT	Mean P-wave amplitude (mV)
	r_wave_amplitude_v5	FLOAT	R-wave amplitude in lead V5 (mV)
	st_segment_elevation	FLOAT	ST segment elevation (mV)
	cardiac_axis	FLOAT	Frontal plane cardiac axis (degrees)
	rmssd	FLOAT	Heart-rate variability: root mean square of successive differences (ms)
	sdnn	FLOAT	Heart-rate variability: standard deviation of NN intervals (ms)
	snr_db	FLOAT	Signal-to-noise ratio (dB)
	is_arrhythmic	BOOLEAN	Automated arrhythmia detection flag
	n_beats_detected	INTEGER	Number of beats detected in the recording
patient_measurements	measurement_id	UUID (PK)	Internal surrogate key
	patient_id	UUID (FK)	References patients.patient_id
	ecg_id	UUID (FK)	References ecg_records.ecg_id
	height_cm	FLOAT	Height in centimetres
	weight_kg	FLOAT	Weight in kilograms
	pectoral_diameter_cm	FLOAT	Pectoral diameter (cm)
	abdominal_diameter_cm	FLOAT	Abdominal diameter (cm)
	physical_status	VARCHAR(100)	General physical status classification
	somatotype	VARCHAR(100)	Body type classification
	position	VARCHAR(100)	Body position during recording (e.g., supine)
	previous_pathologies	TEXT	Prior medical conditions (free text)
	previous_treatments	TEXT	Prior treatments or medications (free text)
medical_users	user_id	UUID (PK)	Internal surrogate key
	email	VARCHAR(255)	Login email address (UNIQUE)
	name	VARCHAR(100)	Given name
	surname	VARCHAR(100)	Family name
	phone	VARCHAR(30)	Contact phone number (nullable)
	institution	VARCHAR(255)	Clinical or academic affiliation (nullable)
	institution_role	VARCHAR(100)	Role within the institution (nullable)
	web_role	VARCHAR(50)	Platform-level access role (nullable)
	specialty	VARCHAR(100)	Medical specialty (nullable)
	license_number	VARCHAR(100)	Professional licence number (nullable)
	country	VARCHAR(100)	Country of practice (nullable)
	profile_image_url	VARCHAR(1024)	Profile image URL (nullable)
	is_active	BOOLEAN	Account status flag
	created_at	TIMESTAMP	Record creation timestamp
	updated_at	TIMESTAMP	Record last-update timestamp (nullable)
user_patients	user_id	UUID (PK/FK)	References medical_users; part of composite PK
	patient_id	UUID (PK/FK)	References patients; part of composite PK
	assigned_at	TIMESTAMP	Timestamp of patient assignment
	access_role	VARCHAR(50)	Access level (primary, consulting, read_only)

The full REST API endpoint catalog

**Table 8:** Complete REST API endpoint catalogue (base path /api/v1) The REST API exposes 46 versioned endpoints grouped by resource; all return JSON and write endpoints accept JSON request bodies. GET, POST, PATCH, and DELETE denote the HTTP methods REST, representational state transfer; API, application programming interface; UUID, universally unique identifier; ECG, electrocardiography

Method	Endpoint	Description
Health		
GET	/health	System and storage health check
Patients		
GET	/patients/	List patients (paginated, with filters)
GET	/patients/count	Total patient count
GET	/patients/by-db-id/{db_patient_id}	Retrieve patient by dataset-specific ID
GET	/patients/{patient_id}	Retrieve patient by internal UUID
GET	/patients/{patient_id}/ecg-records	List ECG records for a given patient
POST	/patients/	Create a patient record
PATCH	/patients/{patient_id}	Partially update a patient record
DELETE	/patients/{patient_id}	Delete a patient record
Patient identities		
GET	/patient-identities/	List identity records
GET	/patient-identities/by-patient/{patient_id}	Retrieve identity by patient UUID
GET	/patient-identities/{identity_id}	Retrieve identity by internal UUID
POST	/patient-identities/	Create an identity record
PATCH	/patient-identities/{identity_id}	Update an identity record
DELETE	/patient-identities/{identity_id}	Delete an identity record
ECG records		
GET	/ecg-records/	List ECG records (paginated, with filters)
GET	/ecg-records/count	Total ECG record count
GET	/ecg-records/without-metrics	Records without computed signal metrics
GET	/ecg-records/without-metrics/count	Count of records without metrics
GET	/ecg-records/by-patient-name	Search records by patient name (VRCardio-Explore)
GET	/ecg-records/{ecg_id}	Retrieve a single ECG record
GET	/ecg-records/{ecg_id}/download-url	Generate presigned Cloudflare R2 download URL
POST	/ecg-records/	Create an ECG record
PATCH	/ecg-records/{ecg_id}	Update an ECG record
DELETE	/ecg-records/{ecg_id}	Delete ECG record and associated R2 file
ECG metrics		
GET	/ecg-metrics/	List ECG signal metrics
GET	/ecg-metrics/count	Total metrics record count
GET	/ecg-metrics/{ecg_id}	Retrieve metrics for a specific ECG record
POST	/ecg-metrics/	Create a metrics record
PATCH	/ecg-metrics/{ecg_id}	Update a metrics record
DELETE	/ecg-metrics/{ecg_id}	Delete a metrics record
Patient measurements		
GET	/patient-measurements/	List anthropometric measurements (with filters)
GET	/patient-measurements/{measurement_id}	Retrieve a single measurement record
POST	/patient-measurements/	Create a measurement record
PATCH	/patient-measurements/{measurement_id}	Update a measurement record
DELETE	/patient-measurements/{measurement_id}	Delete a measurement record
Medical users		
GET	/medical-users/	List registered medical users
GET	/medical-users/by-email	Retrieve user by email address
POST	/medical-users/upsert	Create or update a medical user (upsert)
POST	/medical-users/upload-ecg	Upload ECG file and create associated records
GET	/medical-users/{user_id}	Retrieve a medical user
GET	/medical-users/{user_id}/patients	List patients assigned to a user
POST	/medical-users/{user_id}/assign-patient/{patient_id}	Assign a patient to a user with a specified role
PATCH	/medical-users/{user_id}	Update a medical user record
POST	/medical-users/recover-vrcardio-ecgs	Recover VRCardio-Explore ECG records from R2 storage
DELETE	/medical-users/{user_id}/test-data	Delete test data for a medical user

Complete benchmark results

**Table 9:** Query reformulations used in the robustness analysis Each cluster comprises five linguistically distinct phrasings encoding the same clinical intent. IoU is the mean pairwise intersection-over-union of the retrieved record sets across the five phrasings; a value of 1.00 indicates that all five phrasings returned an identical cohort IoU, intersection-over-union; HR, heart rate; QTc, corrected QT interval; AF/AFIB, atrial fibrillation; ECG, electrocardiography

Cluster	Reformulations	IoU
Demographic	(1) Show me male patients aged over 65; (2) Men aged more than 65 years; (3) Retrieve records for male subjects with age greater than sixty-five; (4) Male patients, age >65; (5) Male sex, older than 65 years old	1.00
ECG thresholds	(1) Patients with heart rate above 100 bpm; (2) Tachycardia, HR >100 bpm; (3) Records where heart rate exceeds one hundred beats per minute; (4) Select patients with elevated heart rate, over 100; (5) HR >100, all sources	1.00
Diagnostic codes	(1) Records with atrial fibrillation; (2) AF patients; (3) AFIB; (4) Patients diagnosed with atrial fibrillation; (5) Show me AF or AFIB records across all sources	0.86
Multicriteria	(1) Males over 65 with tachycardia; (2) Male patients aged more than 65 and heart rate above 100 bpm; (3) Men older than 65 with HR >100; (4) Select male, age >65, heart rate >100; (5) Elderly male patients with heart rate over 100 beats per minute	1.00
Mixed clinical	(1) Elderly patients with prolonged QTc; (2) Age >70 and QTc >440 ms; (3) Patients older than 70 years with corrected QT interval above 440 milliseconds; (4) Older patients, age above 70, prolonged corrected QT over 440; (5) Senior patients (70+) with QTc prolongation exceeding 440 ms	1.00
Overall mean	-	0.97

Expert clinical validation protocol and cardiologist feedback

This appendix documents the structured clinical validation summarized in the Expert Clinical Assessment. The protocol was designed to demonstrate two complementary properties of the VRCardio-Helper-Database natural-language interface: usability (whether cardiologists without SQL expertise can interrogate large ECG repositories effectively) and utility (whether the interface answers genuine cardiovascular research questions). Two senior cardiologists from the Cardiology Department of Hospital Infanta Leonor (Madrid, Spain) completed the protocol independently. Both interacted with the system entirely in Spanish; because language understanding is delegated to a multilingual large language model, the interface accepts queries in any language natively supported by the model, so this Spanish-language session also illustrates the language-agnostic usability of the platform. The queries are translated into English in the tables below for presentation.

Each evaluator worked through two rounds of queries. In the first round, they reformulated, in their own words, a set of shared baseline questions spanning the principal query categories (age-QTc correlation, simultaneous wide-QRS and atrial fibrillation, sex differences in heart rate, prevalence of prolonged QTc by age, and proportion of normal ECGs by decade), preserving the clinical intent of each. In the second round, they posed free-choice research questions arising from their own clinical interests, including questions deliberately probing the boundaries of the data (for example, rare inherited syndromes and findings not encoded as discrete diagnostic labels). For every response, the evaluator recorded the verbatim query, the verbatim answer, and six ratings: five clinical dimensions on a five-point Likert scale (medical correctness, whether the response answered the question, interpretability, real clinical value, and whether important details were missing, reverse-scored), together with an overall quality score on a ten-point scale. The aggregate results are reported in Table 6; the complete per-query ratings for each evaluator are provided in Table 10 and Table 11.

**Table 10:** Per-query ratings, Cardiologist A Queries marked with a dagger returned a transient service-availability message (hosted-assistant timeout) and are excluded from the summary statistics (shown as n/a). Queries were posed in Spanish and translated for presentation MC to MISS are scored 1-5; OVR is scored 1-10 MC, medically correct; AQ, answers the question; INT, interpretable; VAL, real clinical value; MISS, missing important details (reverse-scored, lower is better); OVR, overall quality; n/a: not available; QT, QT interval; QRS, QRS interval; QTc, corrected QT; ECG, electrocardiography

No.	Query (translated from Spanish)	MC	AQ	INT	VAL	MISS	OVR
1	Is there a significant correlation between age and corrected QT?	5	5	5	4	1	8
2	How many patients (count and proportion of the population) have both wide QRS and atrial fibrillation? (dagger)	n/a	n/a	n/a	n/a	n/a	n/a
3	How many patients have both wide QRS and atrial fibrillation?	3	2	2	1	1	3
4	What are the main sex differences in mean heart rate?	5	5	5	5	1	10
5	Prevalence of prolonged QTc by age group? (dagger)	n/a	n/a	n/a	n/a	n/a	n/a
6	Prevalence of prolonged QTc by age?	5	4	5	4	1	8
7	Proportion of ECGs considered normal by age decade?	5	5	5	4	1	8
Free-choice research questions							
8	How is this database composed, and from which populations, countries, and dates do the ECGs come?	5	5	5	5	1	10
9	Who interprets the ECGs: automatic measurement or cardiologist-validated?	4	5	5	5	4	9
10	Proportion of patients with ECGs meeting major/minor diagnostic criteria for ARVD?	5	5	5	5	1	10
11	(follow-up) "Yes, search for them." (dagger)	n/a	n/a	n/a	n/a	n/a	n/a
12	Patients with long-QT syndrome (QTc >500 ms) and suspected cases (QTc >470/480 ms), with age, sex, geography? (dagger)	n/a	n/a	n/a	n/a	n/a	n/a
13	How many patients have QTc >500 ms? Mean age, sex, geography? (dagger)	n/a	n/a	n/a	n/a	n/a	n/a
14	How many patients have QTc >500 ms?	4	5	5	4	2	8
15	Of those with QTc >500 ms, distribution by age range and sex?	4	5	5	3	1	9
16	Could patients with QTc >500 ms and age <45 be located/identified?	5	5	5	2	4	10
17	(Follow-up) "Deepen the clinical analysis of this high-risk group? OK."	1	1	5	1	5	1
18	How many ECGs show a pattern compatible with Brugada syndrome?	1	5	5	1	5	2
19	How many patients have a short PR interval? Distribution by age and sex?	4	5	5	3	3	7
20	Proportion of short-PR ECGs relative to the total per age group?	1	1	1	1	5	1

**Table 11:** Per-query ratings, Cardiologist B Same rating scheme and abbreviations as for Cardiologist A; queries marked with a dagger returned a transient timeout and are excluded from the summary statistics. Queries were posed in Spanish and translated for presentation n/a: not available; QT, QT interval; QRS, QRS interval; ECG, electrocardiography; AV, atrioventricular; ECG, electrocardiography

No.	Query (translated from Spanish)	MC	AQ	INT	VAL	MISS	OVR
1	Is there a correlation between age and QTc?	5	5	5	3	1	9
2	How many patients with wide QRS and atrial fibrillation simultaneously? (dagger)	n/a	n/a	n/a	n/a	n/a	n/a
3	Sex differences in mean heart rate?	5	5	5	3	1	8.5
4	What is the most frequent combination of ECG findings?	4	5	4	2	4	7.5
5	What percentage of ECGs are normal per decade of life?	4	5	5	3	3	7.5
Free-choice research questions							
6	Are there ECG findings associated with the future development of atrial fibrillation on follow-up? (dagger)	n/a	n/a	n/a	n/a	n/a	n/a
7	Most frequent ECG finding in patients with severe valvular disease?	5	4	5	1	1	6
8	Atrial fibrillation prevalence by age?	5	5	5	3	1	9
9	Most characteristic ECG finding by decade of age?	4	5	5	3	1	8
10	How does the PR interval vary by decade of life?	5	5	5	3	1	8
11	Isn't it odd that the median PR is below the normal range in the older population?	5	5	4	2	1	9
12	How can first-degree AV block exist if no patient has PR >200 ms?	5	5	4	2	2	9
13	How does the cardiac axis change with age?	5	5	5	3	1	9
14	Which ECG finding do you consider most clinically important in this database?	5	5	5	4	1	9
15	From what age would you propose atrial-fibrillation screening with 12-lead ECG?	5	5	5	5	1	10

Both evaluators accompanied their ratings with free-text comments; representative comments (translated from Spanish) are reproduced below. The cardiologists repeatedly highlighted the analytical depth, clarity, and clinical framing of the responses. On the sex-difference-in-heart-rate analysis (rated 10/10), one commented: "I was surprised by the analysis it performed and the conclusions it reached. The clinical interpretation and the possible causes are plausible. Very good." On the database-composition query (10/10): "Sufficient information to know the origin of the data and thus assess whether it is extrapolable to the current Spanish population." On the arrhythmogenic-right-ventricular-dysplasia query (10/10): "Correct and as expected. It was a complex clinical question that the database resolves well, and I like that it then offers to search the data another way." On a privacy-aware response to a patient-identification request (10/10): "Correct and expected, given data-protection law and the origin of the ECGs. It could be used for population-level screening and large-scale identification of at-risk patients." On an atrial-fibrillation screening recommendation (10/10): "I really like it, because it goes one step further and proposes, with data, a clinical course of action," noting that the more recent 2024 European Society of Cardiology guidelines on atrial fibrillation are also available [29]. On data-provenance transparency: "It is important for the cardiologist to know who interpreted the ECGs, whether the results are based on automatic interpretations or on assessments by specialists."

A particularly informative exchange demonstrated the interface's capacity for self-critical, methodologically aware analysis. After noticing that the population median PR interval sat at the low-normal boundary and was implausibly low in older patients, one evaluator asked the system to account for the discrepancy. The interface performed a critical re-analysis that correctly identified the cause: PR-interval values were stored in seconds and truncated near 0.150 s by the metric-extraction pipeline, so true first-degree AV block (PR >200 ms) could not be detected from the numeric metric, while the corresponding diagnostic labels remained valid; on that basis it recommended using the diagnostic codes rather than the numeric PR metric for conduction studies. The evaluator commented: "I love the self-criticism it performs." This artifact, consistent with the PR statistics in Table 2, has since been corrected at the metric-extraction stage.

The evaluators also identified concrete improvement opportunities. On service reliability, several of the more complex multi-criteria queries returned a transient "the assistant is busy" message rather than an analysis, in some cases after repeated attempts; the evaluators noted these were precisely the type of question they would naturally pose, and that simplifying or decomposing the query usually elicited a correct response, indicating a concurrency and request-timeout issue at the hosted-assistant layer rather than a semantic limitation. On presentation, for several queries the evaluators preferred prevalence relative to the appropriate denominator over absolute counts. Both evaluators flagged an implausibly low proportion of "normal" ECGs in the 0-9-year age group, correctly hypothesizing that adult ECG-normality criteria had been applied to pediatric recordings, whose normal patterns differ substantially. Comments also noted occasional mixing of English and Spanish terminology and inconsistent font sizing. Finally, on the most-frequent-combination query, one evaluator observed that some co-occurring code pairs are mutually exclusive within a single recording (for example, sinus tachycardia with atrial fibrillation) or warrant clinical caution (for example, simultaneous right and left bundle branch block), highlighting the value of physiology-aware post-processing of multi-label results.

Overall, the structured validation confirms that cardiologists without SQL expertise could pose clinically meaningful research questions in natural language and obtain interpretable, clinically plausible results, while also surfacing a focused set of reliability and presentation refinements that inform the development roadmap.

## References

[1] Wagner P, Strodthoff N, Bousseljot RD, Kreiseler D, Lunze FI, Samek W, Schaeffter T (2020). PTB-XL, a large publicly available electrocardiography dataset. Sci Data.

[2] Strodthoff N, Wagner P, Schaeffter T, Samek W (2021). Deep learning for ECG analysis: benchmarks and insights from PTB-XL. IEEE J Biomed Health Inform.

[3] Gow B, Pollard T, Nathanson LA (2026). MIMIC-IV-ECG: diagnostic electrocardiogram matched subset. PhysioNet.

[4] Hripcsak G, Duke JD, Shah NH (2015). Observational Health Data Sciences and Informatics (OHDSI): opportunities for observational researchers. Stud Health Technol Inform.

[5] Achiam J, Adler S, Agarwal S (2023). OpenAI: GPT-4 technical report. [Preprint]. arXiv.

[6] Katsogiannis-Meimarakis G, Koutrika G (2023). A survey on deep learning approaches for text-to-SQL. VLDB J.

[7] Wornow M, Xu Y, Thapa R (2023). The shaky foundations of large language models and foundation models for electronic health records. NPJ Digit Med.

[8] Oh J, Lee G, Bae S, Kwon JM, Choi E (2023). ECG-QA: a comprehensive question answering dataset combined with electrocardiogram. Adv Neural Inf Process Syst.

[9] Pham HM, Tang J, Saeed A, Ma D (2025). Q-HEART: ECG question answering via knowledge-informed multimodal LLMs. [Preprint]. arXiv.

[10] Koretsky MJ, Willey M, Bianchi O (2025). BiomedSQL: text-to-SQL for scientific reasoning on biomedical knowledge bases. [Preprint]. arXiv.

[11] Zheng J, Zhang J, Danioko S, Yao H, Guo H, Rakovski C (2020). A 12-lead electrocardiogram database for arrhythmia research covering more than 10,000 patients. Sci Data.

[12] (2026). Anthropic: model context protocol (MCP): an open protocol for connecting AI models to external data sources and tools. https://modelcontextprotocol.io.

[13] Lowin J (2026). FastMCP: a fast, Python-native framework for building Model Context Protocol servers and clients. https://github.com/jlowin/fastmcp.

[14] Anthropic Anthropic (2026). The Claude 4 model family (Claude Sonnet 4.6 and Claude Haiku 4.5). https://www.anthropic.com/claude.

[15] (2026). The Gemini model family (Gemini 2.5 Pro and Gemini 2.0 Flash). Gemini.

[16] European Committee for Standardization (CEN) (2026). EN 1064: health informatics - standard communication protocol - computer-assisted electrocardiography. CEN Standard.

[17] Johnson AE, Bulgarelli L, Shen L (2023). MIMIC-IV, a freely accessible electronic health record dataset. Sci Data.

[18] Ribeiro AH, Ribeiro MH, Paixão GM (2020). Automatic diagnosis of the 12-lead ECG using a deep neural network. Nat Commun.

[19] Liu F, Liu C, Zhao L (2018). An open access database for evaluating the algorithms of electrocardiogram rhythm and morphology abnormality detection. J Med Imaging Health Inform.

[20] Avetisyan A, Khachaturov N, Asatryan A, Tigranyan S, Markin Y: ISP ECG delineation dataset (2026). ISP ECG delineation dataset. Zenodo.

[21] Avetisyan A, Khachaturov N, Asatryan A, Tigranyan S, Markin Y (2024). Self-trained model for ECG complex delineation. arXiv.

[22] Kalyakulina AI, Yusipov II, Moskalenko VA (2020). LUDB: a new open-access validation tool for electrocardiogram delineation algorithms. IEEE Access.

[23] Laguna P, Mark RG, Goldberger A, Moody GB (1997). A database for evaluation of algorithms for measurement of QT and other waveform intervals in the ECG. Comput Cardiol.

[24] Spika Tech SL (2026). Spika Tech SL: VR-CARDIO: visualization of biopotentials for cardiology equipment. https://cordis.europa.eu/project/id/190123244.

[25] Spika Tech SL (2026). Spika Tech SL: European patent application EP23382800. European Patent Office. European Patent Office.

[26] Rautaharju PM, Surawicz B, Gettes LS (2009). AHA/ACCF/HRS recommendations for the standardization and interpretation of the electrocardiogram: part IV: the ST segment, T and U waves, and the QT interval: a scientific statement from the American Heart Association Electrocardiography and Arrhythmias Committee, Council on Clinical Cardiology; the American College of Cardiology Foundation; and the Heart Rhythm Society. Endorsed by the International Society for Computerized Electrocardiology. J Am Coll Cardiol.

[27] Mason JW, Ramseth DJ, Chanter DO, Moon TE, Goodman DB, Mendzelevski B (2007). Electrocardiographic reference ranges derived from 79,743 ambulatory subjects. J Electrocardiol.

[28] Tsuji H, Larson MG, Venditti FJ Jr, Manders ES, Evans JC, Feldman CL, Levy D (1996). Impact of reduced heart rate variability on risk for cardiac events. The Framingham Heart Study. Circulation.

[29] Van Gelder IC, Rienstra M, Bunting KV (2024). 2024 ESC guidelines for the management of atrial fibrillation developed in collaboration with the European Association for Cardio-Thoracic Surgery (EACTS). Eur Heart J.

[30] Goldberger AL, Amaral LA, Glass L (2000). PhysioBank, PhysioToolkit, and PhysioNet: components of a new research resource for complex physiologic signals. Circulation.

